# A Deep Learning Model for Preoperative Differentiation of Glioblastoma, Brain Metastasis and Primary Central Nervous System Lymphoma: A Pilot Study

**DOI:** 10.3389/fonc.2022.816638

**Published:** 2022-02-24

**Authors:** Leonardo Tariciotti, Valerio M. Caccavella, Giorgio Fiore, Luigi Schisano, Giorgio Carrabba, Stefano Borsa, Martina Giordano, Paolo Palmisciano, Giulia Remoli, Luigi Gianmaria Remore, Mauro Pluderi, Manuela Caroli, Giorgio Conte, Fabio Triulzi, Marco Locatelli, Giulio Bertani

**Affiliations:** ^1^Unit of Neurosurgery, Fondazione IRCCS Cà Granda Ospedale Maggiore Policlinico, Milan, Italy; ^2^Department of Oncology and Hemato-Oncology, University of Milan, Milan, Italy; ^3^Department of Paediatric Orthopaedics and Traumatology, ASST Centro Specialistico Ortopedico Traumatologico Gaetano Pini-CTO, Milan, Italy; ^4^Department of Neurosurgery, Fondazione IRCCS Istituto Neurologico Carlo Besta, Milan, Italy; ^5^Department of Neurosurgery, Trauma Center, Gamma Knife Center, Cannizzaro Hospital, Catania, Italy; ^6^National Center for Disease Prevention and Health Promotion, Italian National Institute of Health, Rome, Italy; ^7^Unit of Neuroradiology, Fondazione IRCCS Cà Granda Ospedale Maggiore Policlinico, Milan, Italy; ^8^Department of Pathophysiology and Transplantation, University of Milan, Milan, Italy; ^9^Aldo Ravelli” Research Center for Neurotechnology and Experimental Brain Therapeutics, University of Milan, Milan, Italy

**Keywords:** brain metastases, deep learning, glioblastoma, machine learning, primary central nervous system lymphoma (PCNSL), artificial intelligence

## Abstract

**Background:**

Neuroimaging differentiation of glioblastoma, primary central nervous system lymphoma (PCNSL) and solitary brain metastasis (BM) remains challenging in specific cases showing similar appearances or atypical features. Overall, advanced MRI protocols have high diagnostic reliability, but their limited worldwide availability, coupled with the overlapping of specific neuroimaging features among tumor subgroups, represent significant drawbacks and entail disparities in the planning and management of these oncological patients.

**Objective:**

To evaluate the classification performance metrics of a deep learning algorithm trained on T1-weighted gadolinium-enhanced (T1Gd) MRI scans of glioblastomas, atypical PCNSLs and BMs.

**Materials and Methods:**

We enrolled 121 patients (glioblastoma: n=47; PCNSL: n=37; BM: n=37) who had undergone preoperative T1Gd-MRI and histopathological confirmation. Each lesion was segmented, and all ROIs were exported in a DICOM dataset. The patient cohort was then split in a training and hold-out test sets following a 70/30 ratio. A Resnet101 model, a deep neural network (DNN), was trained on the training set and validated on the hold-out test set to differentiate glioblastomas, PCNSLs and BMs on T1Gd-MRI scans.

**Results:**

The DNN achieved optimal classification performance in distinguishing PCNSLs (AUC: 0.98; 95%CI: 0.95 - 1.00) and glioblastomas (AUC: 0.90; 95%CI: 0.81 - 0.97) and moderate ability in differentiating BMs (AUC: 0.81; 95%CI: 0.70 - 0.95). This performance may allow clinicians to correctly identify patients eligible for lesion biopsy or surgical resection.

**Conclusion:**

We trained and internally validated a deep learning model able to reliably differentiate ambiguous cases of PCNSLs, glioblastoma and BMs by means of T1Gd-MRI. The proposed predictive model may provide a low-cost, easily-accessible and high-speed decision-making support for eligibility to diagnostic brain biopsy or maximal tumor resection in atypical cases.

## Introduction

Brain metastases (BM), glioblastomas and primary central nervous system lymphomas (PCNSL) are amongst the most common intracranial neoplasms in adults (17%, 14.6%, and 1.9% respectively) (1, 2). Treatments and prognoses differ, and accurate diagnosis is crucial to guide management strategies. Current guidelines suggest maximal surgical resection plus chemoradiation therapy for BMs and glioblastoma and methotrexate-chemotherapy plus whole-brain radiotherapy for PCNSLs (3–6). Biopsy, especially stereotactic, is the diagnostic gold- standard, but the overall complication rate is up to 13% (7). In addition, the use of pre-operative steroids in patients with BMs and glioblastomas, aimed at relieving symptoms, may hinder histopathological diagnoses in PCNSLs, leading to higher false-negative rates (8).

Conventional Magnetic Resonance Imaging (MRI) assists the preoperative diagnostic assessment and guides treatment planning, but lesions may show overlapping radiological features. On T1-weighted gadolinium-enhanced (T1Gd) images, glioblastomas often show peripheral rims of contrast-enhancement and central necroses similar to solitary BMs, whereas PCNSLs frequently exhibit homogeneous enhancement (9, 10). In atypical cases, glioblastomas may display minimal or absent necroses and PCNSLs may show central necroses mimicking glioblastomas (11). Some advanced MRI techniques may support the radiological assessment, for instance by differentiating reduced cerebral blood volume (CBV), characteristic of PCNSLs, from high CBV, frequently reported in glioblastomas (12, 13). However, uncommon hypervascular PCNSLs may be encountered, posing additional diagnostic challenges despite the use of advanced multiparametric imaging. Finally, advanced MRI protocols require greater expertise and expenses, affecting their worldwide applicability (14).

Radiomics has been adopted in neuro-oncology for diagnostic classification and prognostic prediction from the analysis of textural or handcrafted radiological features (15). However, it needs lengthy and meticulous preprocessing steps such as imaging segmentation, manual features selection and extraction. More recently, the introduction of machine learning algorithms significantly improved classification performances (16–18): deep learning methods, in particular deep neural networks (DNN), may automatically perform several computer visions tasks by extracting information directly from radiological sequences (19, 20).

In this study we evaluated the discriminative ability of a deep learning algorithm trained on T1-weighted gadolinium-enhanced (T1-Gd) MRI scans to differentiate glioblastomas, atypical PCNSLs, and BMs, and to improve both diagnostic and interventional workflows.

## Material and Methods

### Patient’s Selection

Ethical approval was waived by the local Ethics Committee in view of the retrospective nature of the study and all the procedures being performed were part of the routine care. Informed consent was obtained from all individual participants included in the study. All procedures performed in studies involving human participants were in accordance with the ethical standards of the institutional and/or national research committee and with the 1964 Helsinki declaration and its later amendments or comparable ethical standards.

We retrospectively reviewed the records of 254 consecutive patients with histologically confirmed glioblastoma, PCNSL with atypical features (immunocompetent patients, central necrotic core, no perivascular location/atypical anatomical location according to the literature or increased rCBV) or BM, who underwent preoperative brain MRI between June 2015 and April 2021. Exclusion criteria included: 1) patients with absent or inadequate MR images; 2) patients with previous intracranial intervention (surgical intervention, gamma knife surgery, or radiation therapy) and 3) the presence of multiple enhancing lesions. Adult immunodeficiency syndrome-related or Epstein-Barr virus-related PCNSL were excluded from our analysis as both subtypes of PCNSL might have increased the heterogeneity of the population investigated.

Following these criteria 121 patients were selected and split, following a 70/30 ratio, into a training set, for deep learning model training, and a balanced hold-out test set, to internally validate the developed DNN model.

### MR Acquisition and Image Preprocessing

All brain MRI studies were performed with a 3 Tesla scanner (Philips® Achieva, Eindoven, Netherlands) using a conventional 32-channel head coil. The protocol included: axial T2-weighted (T2w) sequence, three-dimensional (3D) Fluid Attenuated Inversion Recovery (FLAIR) sequence, axial diffusion-weighted images (DWI) with b-values of 0-1000 sec/mm2 and contrast-enhanced (Gadovist 0.1 mL/kg; Prohance 0.2 mL/kg) axial and three-dimensional (3D) T1-weighted sequences.

**Figure 1:**
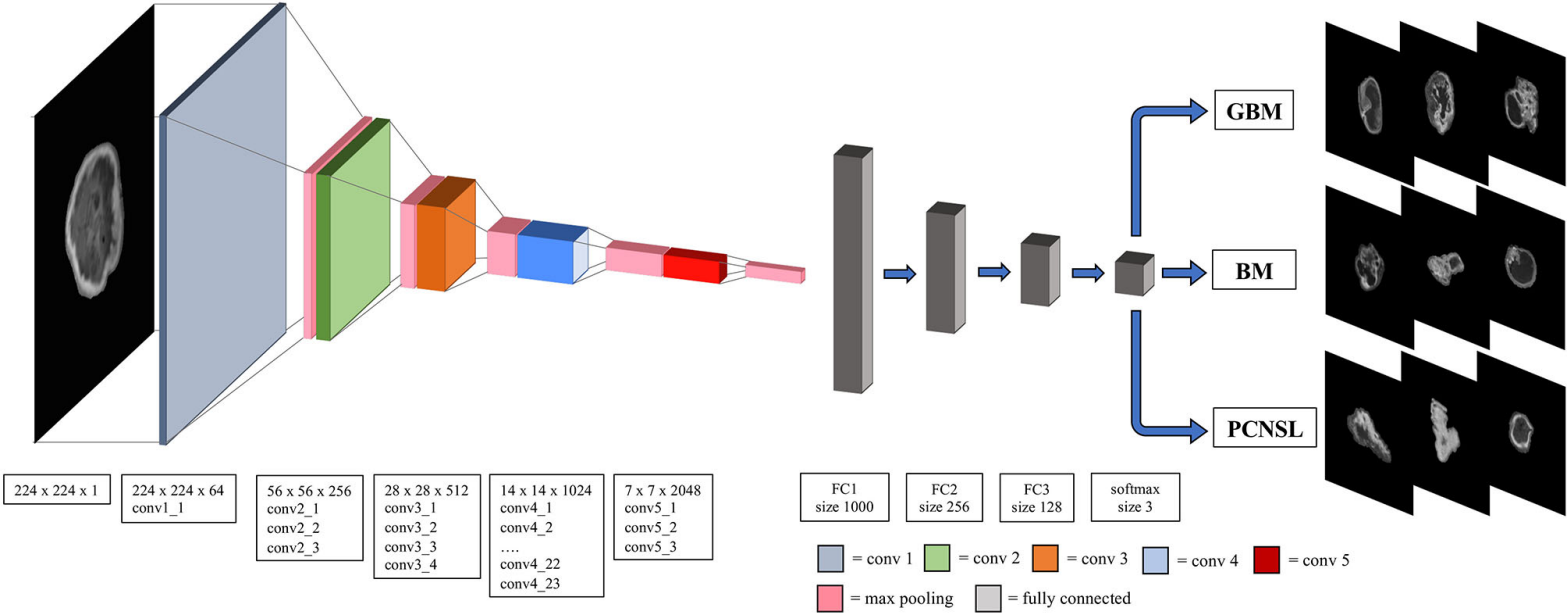
The overall Resnet-101 model architecture. The window size and the stride for convolutional, maxpooling and fully connected layers are also presented. Conv, convolutional layer; FC, fully connected layer; GBM, glioblastoma multiforme; PCNSL, primary central nervous system lymphoma; BM, brain metastasis.

All MR images in the form of digital imaging and communications in medicine (DICOM) were input to the Horos DICOM Viewer (version 3.3.5, www.horosproject.org) a free, open-source medical imaging viewer and analytic tool. Using this software, the regions of interest (ROIs) of these three types of lesions were manually delineated on every section in which the tumoral mass was visualized on preoperative axial CE-T1. After volume acquisition, segmentation and signal intensity normalization, all the ROIs were then centered in a 224x224 pixels black box and exported in PNG file format (**Figure 1**).

### Convolutional Neural Network Model

A 2D convolutional neural networks (specifically the Resnet-101 model) with 101 layers consisting of 3-layer residual blocks pre-trained with the ImageNet database was used (21, 22). Hyperparameters of the fully connected layer of ResNet were fine-tuned with the training set data while the convoluting and pooling layers were frozen in order to preserve the features extraction capability of the pretrained Resnet-101 model. The batch size was 32, and a drop-out rate of 0.25 was applied with rectifier linear unit as the activation function to minimize model overfitting. The model was trained for 50 epochs with stochastic gradient descent optimized with the Adam optimizer and the initial learning rate set to 0.005 (23). Batch normalization was used in each layer to improve learning stability (24). The structure of the developed DNN model is depicted in **Figure 1**.

**Figure 2:**
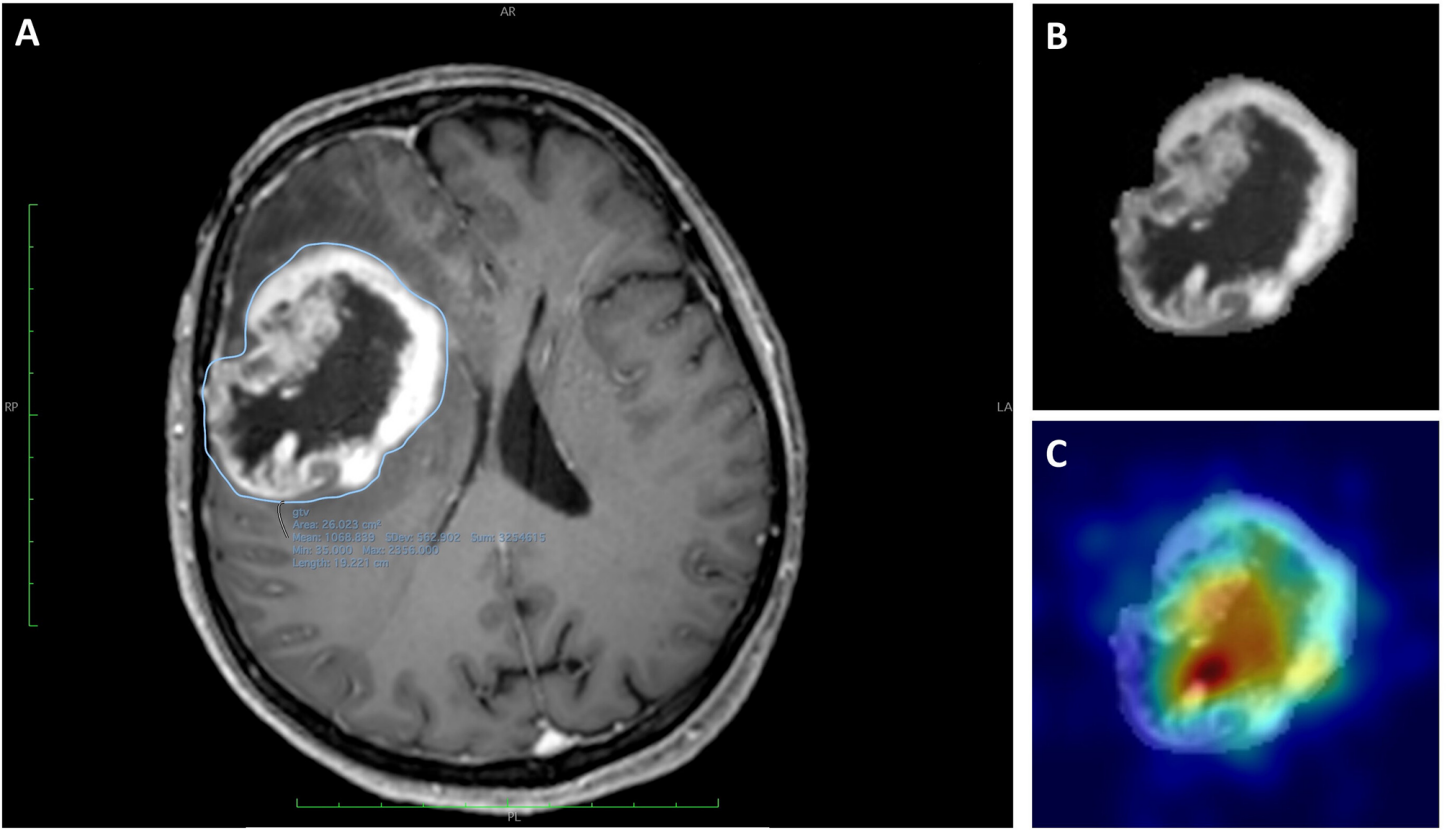
Images of a 59-year-old woman with a histologically diagnosed GBM. **(A)** CE T1WI with the segmented region of interest (ROI). **(B)** Extracted ROI of the segmented lesion. **(C)** Extracted ROI and the corresponding gradient-weighted class activation map (Grad-CAM) showing the salient tumor regions identified by Resnet-10 and on which the DNN model relies to make its prediction.

Each ROI was used as inputs for all the 3 channels expected by the Resnet model and was treated as an independent image to increase the number of input data even though a group of slices belonged to the same patient. However, to prevent data leakage from the training to the hold-out test set, data splitting during training of the model was done per patient and not per section image. The predicted diagnostic class for each patient was the most frequently voted one among its entire ROIs set. Gradient-weighted class activation mapping (Grad-CAM) visualization was used to evaluate which portion of the tumoral lesion the developed DNN model was focusing on in order to make each patient’s diagnostic prediction (an example is reported in **Figure 2**) (25).

The final model was internally validated on the hold-out test set. The performance metrics reported were computed considering the number of mis considered per patient and not per image.

### Performance Metrics

The classification performance of the DNN model was evaluated considering the following performance metrics: 1) Area Under the Receiving Operative Characteristics curve (AUC-ROC); 2) Accuracy; 3) Precision or Positive predictive value (PPV); 4) Negative predictive value (NPV); 5) Recall or sensitivity; 6) Specificity; 7) F-1 score.

A One-vs-the-Rest (OvR) multiclass strategy was employed to extract performance metrics for each outcome class, then the average value and its 95% bootstrap confidence interval were computed for each of the above-mentioned performance metrics on the hold-out test set. A grouped binary comparison was also performed in order to investigate the reliability of the DNN model in distinguishing surgically resectable lesions (glioblastoma or BM) from non-resectable ones (PCNSL).

Finally, the retrospective diagnostic performance of neuroradiologists with at least 10 years of dedicated experience reviewed by means of radiological report charts was computed and defined as “gold standard” for comparison with the DL model.

### Statistics, Software and Hardware

Descriptive statistics, frequencies and percentages were used to report tumor volume characteristics. A Shapiro-Wilk normality test was used to assess normality. When appropriate, continuous variables were reported as mean + standard deviation (SD) or median and interquartile range (IQR). Statistical differences in tumor volumes were tested using the ANOVA or Kruskall Wallis test, according to normality of the sample. All the statistical analyses were performed in Jupyter Notebook, using Python v.3.7.6 (https://www.python.org/). The Python packages used for this study included: ‘PyTorch v1.7’ to develop and train the DNN model, ‘Numpy’ for Excel dataset handling; ‘Scikit-learn’ to compute performance metrics and ‘Seaborn’ to plot ROC-AUC. The workstation used to train the DNN model mounted an Intel Core i7-10700K processor while the GPU was a Tesla K80 12GB.

## Results

The cohort of selected patients included: 47 glioblastomas (age: 61.3 [48.9-73.7] years), 37 PCNSLs (age: 51.1 [43.3-58,9] years) and 37 BMs (age 59.5 [49.9-69.1] years). The male-to-female ratio was 50/71 (58.3% were female). Median tumor volumes were as follows: glioblastoma (56.31[45.50-69.00], PCNSL (39.00 [31.40-45.25] and BM (56.50 [44.01-65.25].A statistically significant different in tumour volume was found (p=0.03). A total of 3’597 axial slices/ROIs of tumors were extracted from 121 patients with: glioblastoma (1’481 ROIs), PCNSL (1’073 ROIs) and BM (1’043 ROIs). No significant difference in age and sex distribution was found between the three groups of patients. Patients in the metastasis group included those with various primary tumor subtypes: 14 (37.8%) lung cancers, 9 (24.3%) breast cancers, 6 (16.2%) colorectal cancers, 5 (13.5%) melanomas and 3 (8.1%) endometrial cancers.

### DNN Model Performance Metrics Evaluation

The trained DNN model, evaluated on the hold-out test set, achieved an AUC of 0.98 (95%CI: 0.95 - 1.00), 0.90 (95%CI: 0.81 - 0.97), 0.81 (95%CI: 0.70 - 0.95) respectively for PCNSL, glioblastoma and BM diagnostic class demonstrating high discriminative ability. High reliability was reported across all performance metrics for atypical PCNSL and moderate for glioblastoma and BM (**Table 1**)

**Table 1:** Convolutional neural networks model’s performance metrics in differentiating PCNSL, Glioblastoma, and BM.

Performance Metrics	PCNSL	Glioblastoma	BM
**AUC**	0.98 (0.95 - 1.00)	0.90 (0.81 - 0.97)	0.81 (0.70 - 0.95)
**Accuracy**	94.65% (89.19% - 100.00%)	83.08% (72.83% - 91.89%)	81.07% (70.27% - 91.89%)
**Precision (PPV)**	91.57% (76.92% - 100.00%)	75.50% (66.16% - 92.31%)	71.11% (59.42% - 93.7%)
**Negative predictive value (NPV)**	93.54% (87.38% -100.00%)	79.92% (69.34%-91.45%)	84.37% (74.78% - 94.15%)
**Recall (Sensitivity)**	91.03% (75.73% - 100.00%)	80.01% (71.23% - 100.00%)	63.61% (53.36% - 82.91%)
**Specificity**	96.23% (88.46% - 100.00%)	81.84% (69.18% - 95.45%)	88.46% (76.92% - 95.31%)
**F1-Score**	0.91 (0.84 - 1.00)	0.77 (0.69 - 0.91)	1.68 (0.54 - 0.83)

Performance metrics achieved by the trained CNN model on the hold-out test set were computed adopting a One-vs-Rest (OVR) multiclass strategy. Average value and 95% bootstrap confidence interval are reported. PCNSL, primary central nervous system lymphoma; BM, brain metastasis; AUC, area under the curve; PPV, positive predictive value; NPV, negative predictive value.

In fact, the model reported 91.57% (76.92% - 100.00%) and 93.54% (87.38%-100.00%) PPV and NPV for PCNSL, respectively, confirming high reliability in ruling in and out candidates in a population-based scenario. By reviewing the capacity to detect BM among all cases, the model was found to be highly reliable in excluding the suspect of BM in favor of a different prediction outcome than confirming it [specificity: 88.46% (76.92% - 95.31%); NPV: 84.37% (74.78%-94.15%)]. Finally, a moderate absolute (sensitivity: 80.01% (71.23% - 100.00%); specificity: 81.84% (69.18% - 95.45%)) and clinical performance was reported for glioblastomas among the cases the model was tested on.

**Figure 3:**
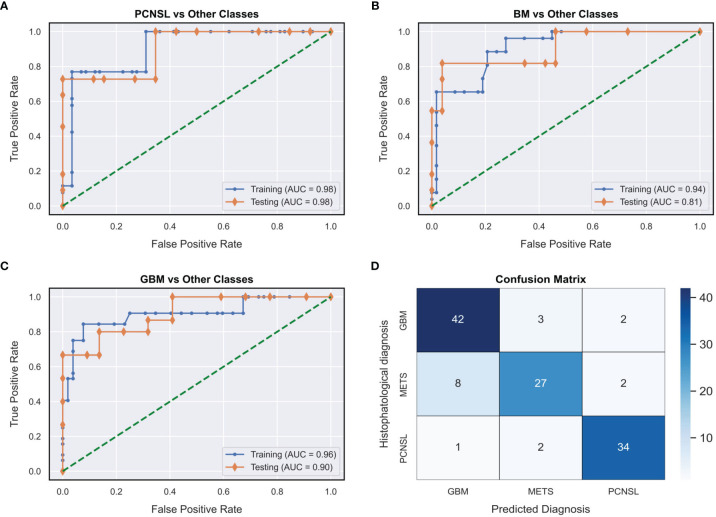
AUC-ROC curves (on both training and hold-out test sets) for each diagnostic outcome class [One-vs-Rest: **(A–C)**] and global confusion matrix **(D)**. GBM, glioblastoma; PCNSL, primary central nervous system lymphoma; METS, brain metastasis.

Moreover, the trained DNN model achieved an AUC of 0.92 (95%CI: 0.83 - 0.99) and an accuracy of 94.7% (95%CI: 89.19% - 100.0%) in distinguishing surgically resectable lesion (glioblastoma or BM) from non resectable ones (PCNSL) (**Table 2**). For each diagnostic outcome class the AUC-ROC curves, achieved on both training and hold-out test set, and the global confusion matrix are depicted in **Figure 3**.

**Table 2:** Convolutional neural networks model’s performance metrics in suggesting lesion resectability.

Performance Metrics	RESECTABLE (GBM or BM) *vs* NON-RESECTABLE (PCNSL)
**AUC**	0.92 (0.83 - 0.99)
**Accuracy**	94.72% (89.19% - 100.0%)
**Precision (PPV)**	91.88% (78.57% - 100.0%)
**Negative predictive value (NPV)**	94.76% (86.13% -100.00%)
**Recall (Sensitivity)**	90.84% (72.73% - 100.0%)
**Specificity**	96.34% (88.46% - 100.0%)
**F1-Score**	0.91 (0.82 – 1.00)

Performance metrics achieved by the trained CNN model on the hold-out test set in evaluating the resectability of the underlying lesion. Average value and 95% bootstrap confidence interval are reported. PCNSL, primary central nervous system lymphoma; GBM, glioblastoma multiforme; BM, brain metastasis; AUC, area under the curve; PPV, positive predictive value; NPV, negative predictive value.

### DNN Model and Neuroradiological Assessment (Gold Standard) Comparison

As gold standard reference, the classification performance of neuroradiologists with at least 10 years of experience was retrospectively conducted on the same population. Every physician had access to the whole multi-sequence DICOM package and independently classified each tumor according to their knowledge. Overall, an optimal accuracy was reached on each tumor type (atypical PCNSL: 84.38%; glioblastoma: 85.87%; BM: 91.67%). The lowest performance was noted when an atypical PCNL was to identify: the overall sensitivity and specificity were 55.56% and 91.03% (PPV: 58.82%; NPV: 89.87%). Additional information are shown in **Table 3**.

**Table 3:** Neuroradiologists (Gold standard) performance metrics in differentiating PCNSL, Glioblastoma and BM in the cohort examined.

Performance Metrics	PCNSL	Glioblastoma	BM
**Accuracy**	84.38%	85.87%	91.67%
**Precision (PPV)**	58.82%	93.33%	78.26%
**Negative predictive value (NPV)**	89.87%	80.43%	95.89%
**Recall (Sensitivity)**	55.56%	82.35%	85.71%
**Specificity**	91.03%	80.43%	93.33%
**F1-Score**	57.46%	87.50%	81.80%

Performance metrics achieved by neuro-radiologists (defined as the gold standard) adopting a One-vs-Rest (OVR) multiclass strategy. The metrics were retrospectively computed by examining patients report charts: all patients underwent conventional plus advanced (T1-weighted, T2-weighted, FLAIR, diffusion-weighted, conventional T1-contrast-enhanced, dynamic contrast-enhanced and perfusion) MRI scans. Values were reported as single computation, so 95% bootstrap confidence interval were not defined. PCNSL, primary central nervous system lymphoma; BM, brain metastasis; PPV, positive predictive value; NPV, negative predictive value.

The DL model yielded an increase in accuracy of +14% for PCNSL, +5% for glioblastoma and a decrease of 10% for BM compared to the gold standard. According to the performance metrics evaluation, the most reliable prediction computed by the DL model was atypical PCNSL.

## Discussion

In the current study, we demonstrated the feasibility of a deep learning model to differentiate glioblastomas, PCNSLs and BMs in routine clinical settings. We found that our DNN model trained on T1Gd-weighted volumetric MRI axial scans showed 83.08%, 94.65%, 81.07% accuracy rates in differentiating each lesion (glioblastomas, PCNSLs and BMs respectively) against the other two. Our model returned the highest accuracy (94.65%) in identifying PCNSLs against the other classes and moderately high diagnostic accuracy for glioblastomas (83.08%) and BMs (81.07%). Moreover, when asked to define the tumor amenability to maximal safe resection or diagnostic biopsy, the deep learning model returned excellent performance (accuracy: 94.72%) and high reliability (PPV: 91.88%; NPV: 94.76%). The algorithm misclassified BMs more frequently than glioblastomas and PCNSLs, probably due to their higher histological heterogeneity and related variability in radiological features; higher accuracy rates would have been obtained by using a larger dataset.

Finally, when the deep learning model and the gold standard (diagnostic reports by neuroradiologists) were compared and evaluated considering the prevalence of each tumor type in the population investigated, a reliable classification performance of the deep learning algorithm was denoted, especially for PCNSL. The greater discriminative power of neuroradiological reports concerning BMs was unsurprising: access to clinical data, history and multiparametric MR imaging in the real-world clinical practice provided essential information the algorithm had no access to. Glioblastomas were the most represented class in our population (47 vs 37 vs 37 cases) and were more accurately classified by the deep learning model than the gold standard, although these results should be carefully reviewed: in fact, though sensitivity and specificity were comparable between the deep learning model and the reference gold standard, the PPV and the NPV showed slightly lower reliability of these results.

Overall, these findings support the clinical experimentation and applicability of the model in assisting physicians to decide whether to proceed with diagnostic biopsy when PCNSLs are suspected or maximal surgical resection when glioblastomas or BMs are more likely.

No single MRI modality is currently capable of differentiating PCNSLs, BMs, and glioblastomas with absolute accuracy. Recent radiomic studies focused on tumor histology prediction (which showed fair performance rates - up to 75%) reported contradictory results in terms of the most predictive MRI sequences analyzed, limiting their applicability in routine clinical practice (26, 27). Fruehwald-Pallamar et al. (28) found that T2-weighted images were more predictive than FLAIR or T1-weighted scans in differentiating benign from malignant tumors. In contrast, Tiwari et al. (29) and Xiao et al. (30) argued that T1Gd images might be superior by showing distinct borders of contrast-enhancing tumors, increasing the accuracy of ROIs segmentation compared to the unclear borders exhibited in T2-weighted and FLAIR scans. Finally, although advanced MRI techniques may improve classification and differentiation of suspected brain neoplasms, their diagnostic role is limited by the operator-dependent interpretation bias, the high heterogeneity among brain tumors and the additional hardware and set-up protocols required, which are available only at major institutions (31, 32).

Previous studies investigated the role of machine learning models to differentiate glioblastomas from PCNSLs. Kunimatsu et al. (27) developed a support vector machine, which, by analyzing radiomic features, returned a 0.75 accuracy in classifying glioblastomas vs PCNSL. Likewise, Xia et al. (33) designed a deep learning algorithm capable of classifying glioblastomas and PCNSLs from multiple MRI sequences with moderate outcomes (accuracy: 0.884). However, no machine learning models aimed at differentiating glioblastomas, PCNSLs and BMs have been reported yet.

Our deep learning algorithm detected with high discriminative capacity specific microscopic parameters of glioblastomas, PCNSLs and BMs and hidden radiological differences between brain tumors. The rationale behind the use of T1Gd images stems from their superior distinction of tumors borders and clear representation of central necroses, which are pathological hallmarks of most glioblastomas and BMs (34, 35).

The segmentation workflow represents a critical aspect of machine learning and deep learning models’ development. As recent computer-based automated segmentation algorithms need to be clinically validated, manual segmentation is still the current gold- standard, showing overall satisfactory results at the cost of intensive work, task-induced fatigue, and extensive processing time. In a recent study, McAvoy et al. (36) developed an EfficientNetB4 DNN with high classification performance for glioblastoma (accuracy: 0.94) vs PCNSL (accuracy: 0.95) on whole brain scan analysis with no prior image segmentation. The authors advocated the superiority of their model compared to previous machine learning studies, as the overall preprocessing effort was sharply reduced. However, the use of non-segmented whole-brain scans may lead to additional classification bias, as DNNs might learn to accomplish their classification task by relying on features (e.g., anatomical location and laterality) determined by unbalanced and heterogeneous training sets instead of clinically related radiological differences, hence limiting the general applicability of the model (37). On the contrary, lesion segmentation partitions each selected slice into a coherent region of interest (ROI) that is extracted from the background and individually processed to acquire overall lesion’s characteristics (either ROI or boundaries). Hence, in our investigation, trained personnel performed manual tumor segmentation.

In 2012 the MICCAI-Brain Tumor Segmentation Challenge (BRATS) (38) was intended to collect the best performing automated segmentation algorithms for brain tumors. The winning algorithm reported high performance in glioma segmentation, but these results are still experimental as automated tools might tend to overestimate volumes and suffer from gross accuracy errors in delineating tumor boundaries (39, 40). In addition, the inclusion of different tumor types in our study would have required several automated segmentation algorithms bearing different and incomparable segmentation performances, introducing larger biases in our training and validation sets than manual segmentation.

We propose the first combined diagnostic “next-move” support tool to assist neuroradiologists in differentiating atypical tumor cases, and neurosurgeons in surgical decision-making processes between resection or biopsy. We trained our model, a low-cost decision-making support solution with extremely high computation speed (within 10 seconds/patient), on a large dataset of conventional T1Gd scans, enabling wider clinical implementations even within institutions with limited resources and restricted access to advanced MRI modalities. Finally, our study design for DNN training and internal validation was built on open-source python packages, and our methodology could be reproduced and externally validated with image datasets from other institutions.

Our study design has some limitations. The number of included patients is larger than most other studies but remains relatively limited and might not address the vast heterogeneity of radiological features that glioblastomas, PCNSLs and BMs exhibit in real clinical settings. Indeed, the limited sample size resulted from selection and inclusion of selected radiologically atypical tumors. We performed a monocentric analysis of images acquired with a specific MRI scanner: for this reason, a validation at different institutions must be run to test model generalizability.

The deep learning architecture itself might introduce some downsides. First, generalizability is often mislead when input data comes from different machines (vendor, models, protocols etc.), as different parametrizations of the input data might alter patterns of deep learning as a consequence of data distribution shift: to minimize this limit, all patients underwent imaging acquisition with a standardized protocol using the same MR scan. Finally, several authors recognized the implicit “black-box” computation as a methodological limitation restraining wider application in clinical practice. In the current study, we deployed a “human-intelligible” visualization method – the Grad-CAM algorithm – to overcome this drawback. However, additional intelligible algorithms have been proposed and comparison of the latter was beyond the scope of the current investigation.

In this study, we trained and internally validated a deep learning algorithm to differentiate atypical and radiologically overlapping cases of glioblastomas, PCNSLs and BMs on T1Gd sequences. A secondary analysis to define the best next-step intervention was conducted with outstanding performance. Other than externally validate our findings, further investigation should prospectively compare the diagnostic and management performances of neuroradiologists and neurosurgeons whether they implement our DL algorithm or not. The proposed model provides a low-cost, easily accessible and high-speed decision-making support for eligibility to diagnostic brain biopsy or maximal tumor resection in atypical tumor cases.

## Data Availability Statement

The raw data supporting the conclusions of this article will be made available by the authors, without undue reservation.

## Ethics Statement

Ethical review and approval was not required for the study on human participants in accordance with the local legislation and institutional requirements. The patients/participants provided their written informed consent to participate in this study.

## Author Contributions

Conceptualization: LT, VC, and ML. Methodology: LT, VC, GF, LM, GCo and LS. Formal analysis and investigation: LT and VC. Writing—original draft preparation: LT, VC, MG, PP, GR, and GB. Writing—review and editing: MG, GB, SB, MP, MC, GR, GCo, GCa, and ML. Resources: GCo, ML, and FT. Supervision: ML, FT, GB, and GCo. All authors contributed to the article and approved the submitted version.

## Funding

This study was supported by the Italian Ministry of Health (Ricerca Corrente 2022).

## Conflict of Interest

The authors declare that the research was conducted in the absence of any commercial or financial relationships that could be construed as a potential conflict of interest.

## Publisher’s Note

All claims expressed in this article are solely those of the authors and do not necessarily represent those of their affiliated organizations, or those of the publisher, the editors and the reviewers. Any product that may be evaluated in this article, or claim that may be made by its manufacturer, is not guaranteed or endorsed by the publisher.
